# Quantitative Interpretation of a Genetic Model of Carcinogenesis Using Computer Simulations

**DOI:** 10.1371/journal.pone.0016859

**Published:** 2011-03-08

**Authors:** Donghai Dai, Brandon Beck, Xiaofang Wang, Cory Howk, Yi Li

**Affiliations:** 1 Department of Obstetrics & Gynecology, University of Iowa, Iowa City, Iowa, United States of America; 2 Department of Mathematics, University of Iowa, Iowa City, Iowa, United States of America; University of Georgia, United States of America

## Abstract

The genetic model of tumorigenesis by Vogelstein et al. (V theory) and the molecular definition of cancer hallmarks by Hanahan and Weinberg (W theory) represent two of the most comprehensive and systemic understandings of cancer. Here, we develop a mathematical model that quantitatively interprets these seminal cancer theories, starting from a set of equations describing the short life cycle of an individual cell in uterine epithelium during tissue regeneration. The process of malignant transformation of an individual cell is followed and the tissue (or tumor) is described as a composite of individual cells in order to quantitatively account for intra-tumor heterogeneity. Our model describes normal tissue regeneration, malignant transformation, cancer incidence including dormant/transient tumors, and tumor evolution. Further, a novel mechanism for the initiation of metastasis resulting from substantial cell death is proposed. Finally, model simulations suggest two different mechanisms of metastatic inefficiency for aggressive and less aggressive cancer cells. Our work suggests that cellular de-differentiation is one major oncogenic pathway, a hypothesis based on a numerical description of a cell's differentiation status that can effectively and mathematically interpret some major concepts in V/W theories such as progressive transformation of normal cells, tumor evolution, and cancer hallmarks. Our model is a mathematical interpretation of cancer phenotypes that complements the well developed V/W theories based upon description of causal biological and molecular events. It is possible that further developments incorporating patient- and tissue-specific variables may build an even more comprehensive model to explain clinical observations and provide some novel insights for understanding cancer.

## Introduction

Efforts to understand cancer have continued to intensify since the start of a presidential campaign to conquer cancer in 1971 [1]. Mortality rates from cancer remains stubbornly high with more than half a million deaths in the US alone in 2009 [2]. Cancer is considered to be predominantly a genetic disease [3]. It is believed that multiple sequential mutations induce malignant transformation of a normal cell into the cancer founder cell, which then multiplies and evolves to become a clinically detectable tumor [4], [5]. This genetic model of carcinogenesis (referred to here as V theory) is augmented by the elegant description of major cancer features by Weinberg et al which is recognized as a seminal and most comprehensive molecular definition of cancer [3], [6]. We specify these two seminal models collectively as the V/W theory of cancer and have sought to develop a mathematical model capable of quantitatively interpreting the V/W theory.

Epithelial tissues are the most common locus of oncogenesis. However, individual epithelial cells are in a constant developmental process of tissue regeneration, namely from stem cell to proliferating/differentiating cell and, finally, to senescent cells [7]. The short lifetime and continued proliferation of epithelial cells in a tissue with a population of 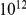 cells pose numerous challenges to determining the natural course of oncogenesis. We have, therefore, attempted to describe the life cycle of an epithelial cell clone of endometrial origin as a normal physiological process to serve as a basic reference for oncogenesis, which is made possible by the addition of many genetic and environmental factors.

### Clone lifetimes during normal epithelial cell regeneration and some major assumptions for the study of carcinogenesis

Endometrial cancer arises in the uterine epithelium, which even in adults are undergoing constant turnover. The tissue stem cells provide a stable cell source for tissue regeneration [7]. A stem cell produces a progenitor cell committed to proliferation, resulting in a clone with hundreds of descendant cells through many generations of cell division. If we assume that the tissue stem cell pool will provide as many progenitor cells as it needs at any time to ensure tissue homeostasis (a stable total cell number), the life span of a clone, from zero cell number (before the birth of a progenitor cell) to one progenitor, to hundreds of descendant cells, to senescence and eventually death, is a cycle from zero cells at the beginning to zero cells at the end over a short time period, days or months. A mathematical description of normal tissue regeneration may identify immortalization (defying programmed senescence and cell death) as an early deviation from the physiological process with a potential for oncogenesis as a result from a combined effect of genetic alterations and environmental stimulations [8]. It thus creates mathematically a continuous and wide spectrum of physiological and pathological cellular events with cancer at the other end. The progenitor cell immediately born from a stem cell and its descendant cells are defined as a **clone** and the entire process (from 0 to 0) is defined as the **clone lifetime**. Thus, any non-stem cell can be tagged and quantitatively analyzed according to chronological time during a clone lifetime. Stem cells are excluded for calculation since their number is maintained through symmetric and asymmetric divisions in the stem cell compartment. For uterine epithelium, there are millions of clones which are actively cycling at any moment but at different stages of their clone lifetime. This ensures a stable total cell number in a tissue for tissue homeostasis.

The progression of a clone lifetime from the progenitor cell is not only shown in the increase of cell number, but also in accompanying differentiation. We derive a mathematical expression to describe the kinetics of a single cell during a clone lifetime. Consequently, a tissue or any population of cells could be treated as a composite of individual cells with cell-specific variables.

Cellular proliferation can be described in terms of density-dependent growth 
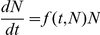
 for some rate function 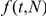. Tumor growth is often studied in the context of Gompertzian growth, where 
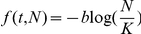
, for constants 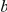 and 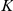 [9], [10]. However, the Gompertz model is empirical and is based on data-fitting with respect to tumor volume and weight for its description of tumor growth [9], [10], although mathematical explanations for the appearance of this growth term have been proposed. In our model we instead consider tumor size as a result of the proliferation of its constituent cells. We derive a set of equations to describe the lifespan of an individual cell within the mass. This process allows us to describe intraclonal heterogeneity in response to mutations and environmental stimulation.

Cellular proliferation is described through a cell's proliferation potential (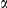, with a unit of doublings per unit time) [11], and occurs by the following: 
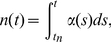
(1)where 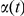 is a cell's proliferation potential and 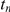 is the time the cell was born. The function 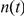 can be interpreted as a measure of a cell's status toward either dividing or dying. If 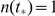 for some time 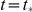 the cell divides, while if 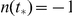 the cell dies. The equation for 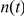 is a consequence of the more common growth terms 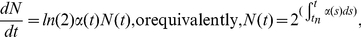
(2)which can be confusing in this context since we are discussing the proliferation of a single cell and not an entire population. The instantaneous 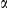 value for any cell in a tumor at any time of its history is measurable theoretically and can be approximated in practice with a mean value which is measured by the change of cell number over a short time period [12]. Hayflick et al described an individual cell's capability to multiply as doubling potential [13]. This concept was further expressed as proliferation potential [14], [15]. It was believed to be critically important to describe intraclonal heterogeneity through description of the difference of doubling potential of individual cells [13]. Consequently, intraclonal heterogeneity can be quantitatively described in a population (a tissue or a tumor) through the summation of individual cells even though all equations here are derived to describe a single cell.

A clone lifetime, starting from a progenitor cell immediately born from a stem cell can be mathematically expressed to capture a dynamic interplay of proliferation and differentiation: 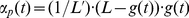
(3)

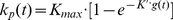
(4)

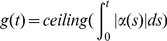
(5)where 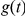 is the generation (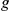) at time 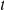 (a quantitative measurement of lineage progression, related to cell division), 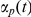 is the generation-dependent programmed (inherent) 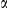, and 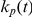 is the corresponding programmed differentiation coefficient (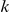). The terms 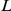, 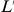, 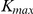 and 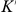 are tissue type-specific constants. Generally, 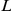 is designated as the maximal programmed generation number before a cell enters a senescent phase according to a cell's inherent developmental program. 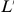 is the tissue-specific conversion coefficient, which converts mathematically the value of generation to that of proliferation potential, dictating the change of programmed proliferation potential value according to a cell's biological clock, a reading of progression of generation. 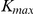 is the coefficient representing the maximal value of differentiation in a tissue for the most mature cell, where greater differentiation implies greater homeostasis according to Assumption 1 below. 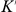 is the respective tissue-specific differentiation conversion coefficient.

Proliferation and differentiation are integrated and continual cell replacement processes in adult tissue homeostasis [7], [16]. In our model, 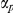 during the developmental process can be described as a gradual increase in its value, reaching the peak at the middle of the developmental stage, and then undergoing a gradual decrease until senescence. Meanwhile, 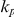 increases gradually and reaches a plateau (maximal level) when cells become well-differentiated. The idea is that cells at the middle of the clone lifetime are the most productive and specialization continues throughout the lifetime. When 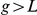, 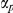 becomes negative, indicating that the cell should enter a process of senescence by its inherent program. This cellular dynamic mimics the gradual proliferation and differentiation process in uterine epithelium during the menstrual cycle.

Here, we must introduce the following assumptions in order to describe the transformation process of oncogenesis.

#### Assumption 1

We hypothesize that one of the most important properties of a normal cell is the maintenance of a programmed proliferation potential 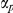 in a developmental stage-specific manner: homeostasis in proliferation or cell number. We define a cell's ability to maintain 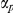 as the resistance potential (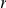) with the following. 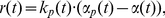
(6)where 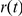 is the resistance potential and 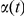 is the proliferation potential at time 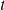. The resistance potential works to bring a cell's 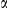 to its programmed level 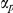, and thus is defined as the force to maintain homeostasis in proliferation. Resistance potential could be executed by cell cycle regulators such as cyclin D1, cdk1, Rb, p16, p27 and p53, one of the most important players capable of arresting cell cycle or triggering apoptosis.

#### Assumption 2

A human cell has in excess of 3 billion base pairs in its genome. Genetic alterations (or mutations), including single and multiple base pair changes, chromosome translocation, aneuploidy and epigenetic alterations, are broadly defined here as any altered hereditary factor, with an enormous number of possibilities. A genetic alteration's effect 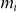 on differentiation status (numerically expressed as differentiation coefficient 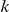) is given as the following: 
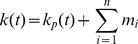
(7)where 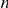 is the number of genetic alterations accrued by the cell during the proliferative process, and 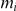 is the numerical value of a genetic alteration, quantifying its effect on the differentiation coefficient. The values 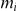 follow a Gaussian distribution with mean 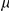 and standard deviation 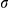 as 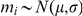. It has been reported that differentiation status of a tumor can be quantitatively defined and tumor subtypes can be classified accordingly [17]. Thus, quantitative change of tumor differentiation should arise from accumulation of mutations according to the V theory of tumorigenesis [4], [5].

#### Assumption 3

A cell lives in a microenvironment filled with various growth and anti-growth signals such as hormones, growth factors, and cytokines. Spatial constraints could have a remarkable effect on cell proliferation as well, especially when a cell is proliferating rapidly (in the case of cancer [18], [19]). The specific effect on a cell by a particular signal is not fixed and not precisely targeted. We assume that the collective effect of all signals on a particular cell follows a Gaussian distribution: 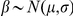. The effect of 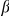 on cell proliferation is expressed by a dynamical change in 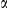. This effect, along with the resistance potential, combines to alter 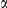 according to: 
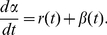
(8)


Instead of combining the assumptions above into a single equation, we present the following four parts collectively as a summary equation for the growth dynamics of an individual cell in order to discuss them more intuitively: 
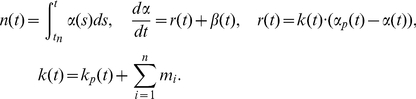
(9)


The clone size is then determined by the summation of all cells within the clone growing according to these dynamics.

## Results

**Figure 1:**
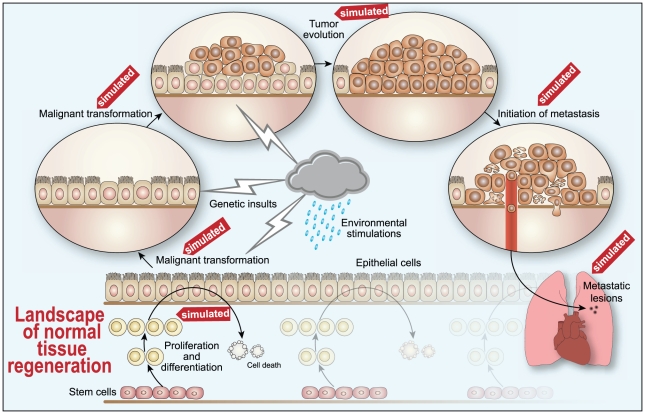
Schematic illustration of some of critical steps of human oncogenesis. These steps are simulated by our model to interpret prevailing genetic theories of cancer. Our model, consisting of a few simple equations, captures the landscape of normal uterine tissue regeneration (simulated for menopausal uterine epithelium and menstrual cycle in Fig. 2), malignant transformation (reduction of differentiation coefficient by genetic alterations in Table 1), occurrence of dormant/transient tumors (simulation of tumor incidence in Tables 2 and 3), tumor evolution (one cascade of tumor evolution resulting in the selection of more aggressive cancer subpopulations in Fig.3), a potential mechanism of the initiation of metastasis (simulated in Fig.4) and the development of clinically detectable metastatic lesions (simulated in Fig. 5). This model describes the evolution of individual cells incorporating quantitative effects from genetic alterations and environment factors, and emphasizes the role of intra-tumor heterogeneity in tumor evolution and metastasis.

In this section, we will explore tissue homeostasis, malignant transformation, tumor evolution, an alternate mechanism for the initiation of metastasis and the survival and establishment of metastatic lesions. The focus will be on endometrial cancer. The mathematical description of normal tissue regeneration is essential to serve as a reference for the process of malignant transformation. Epithelial cell turnover is a dynamic process involving billions of cells and is presented here as a huge landscape with genetic insults (lightning) and environmental stimulations (raining) having an uneven effect on individual cells which will consequently serve as the basis of heterogeneity of oncogenesis and tumor evolution (Fig. 1).

**Figure 2:**
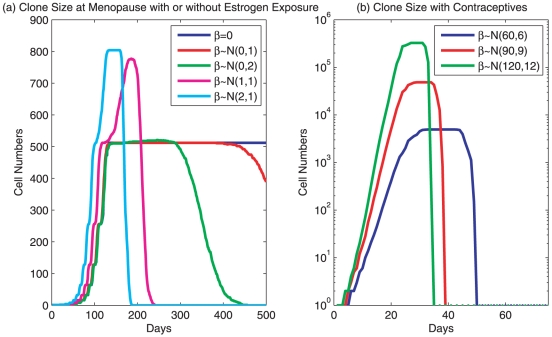
A clone life of the uterine epithelial cells. The life of a clone is perceived to start when a progenitor cell is borne from an asymmetrical division of a stem cell at 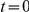. The progenitor cell multiplies to produce the clone (all descendant cells), whose cells exhibit developmental stage specific features described by equations 10, 11 and 5. The clone size is calculated as the summation of all individual cells whose growth is governed by system 9. The size of the entire uterine epithelium at any time will be the summation of all clones with different sizes. A 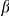 value is assigned to an existing cell every day, randomly and independently. **a. Simulations of postmenopausal endometrium.** 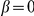 is used to simulate a hypothetical scenario exhibiting no environmental exposure. Neutral 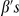, 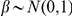 and 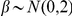, are used as representations of the microenvironmental effect of postmenopause. A small positive 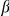, 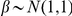 and 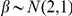, is introduced to reflect a chronic exposure to weak estrogenic stimulation. **b. Simulations of endometrium with monophasic contraceptives.** A monophasic schedule of oral contraceptives is used to model the menstrual cycle. Strong and positive 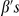, 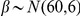, 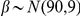, and 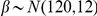, are applied for 21 days in the simulations for the representation of oral estrogen and progestin. The interaction between fast cell growth and microenvironment, and the effect of sudden withdrawal of hormones on blood vessels are not included.

**Figure 3:**
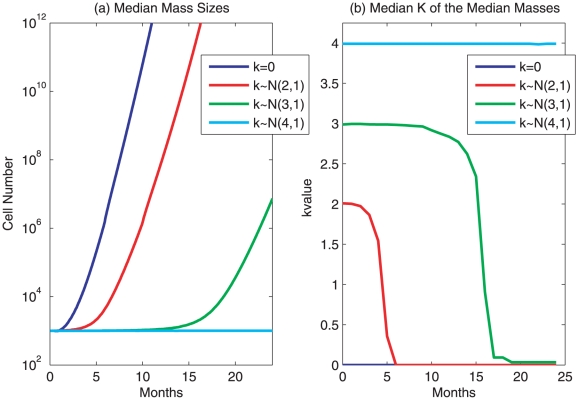
Simulation of tumor evolution. Tumor size change and re-distribution of intra-tumor subpopulations are simulated. **a. The growth curve of four masses with 1000 heterogeneous cells each, specified by different differentiation coefficients to indicate the extent of loss of differentiation.** Every cell has an 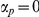 and is assigned at 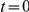, randomly and independently, an 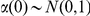 to indicate heterogeneous initial proliferation potentials. Each mass has a 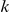 value for its cells from 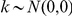, 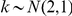, 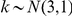, and 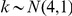, respectively. All cells grow at a neutral condition 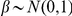. The tumor size is expressed as the total number of cells within a mass, with each individual cell proliferating according to the system 9. A simulation is terminated when the total number of cells exceeds 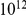. No mutation is considered thus the 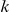 value remains constant for each cell and its descendants. **b. Change of the values of differentiation coefficients over time in the four tumors.** The median 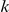 values over time among all the cells of one of 4 masses shown in Fig 3a are shown. The 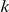 values shown in the legend are the initial distributions of 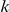 values within each of the four masses at time 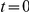.

**Figure 4:**
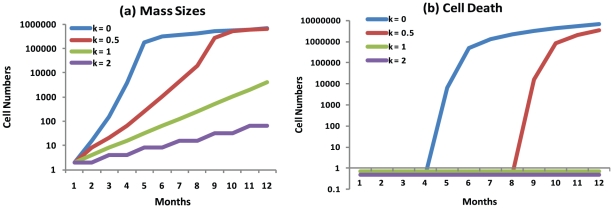
Cell death during tumor progression. Four cells with 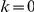, 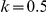, 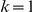 and 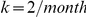, respectively, are growing in an identical growth condition provided by a 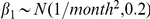, a weak and positive growth stimulation, and an identical initial proliferation potential 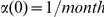. Environmental reaction to tumor growth is based upon 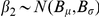, which affects any cell in the basic tissue in a stochastic fashion. The environmental reaction is defined as an ecological balance and is calculated according to equations 12 and 13. No mutation is included, thus the 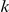 value remains constant for each cell and its descendants in the simulations. **a.** The mass sizes arising from the four cells with different 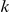 values but identical 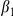 value and initial 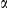 value. **b.** The accumulated numbers of cell death are presented for the four masses with 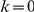, 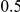, 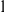 and 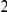, respectively. The accumulated total cell death over 12 months for the cells with 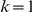 and 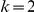 are zero but presented at a value of 0.5 due to the logarithm scale of the y-axis. A 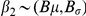 is calculated and assigned to each surviving cell every day.

**Figure 5:**
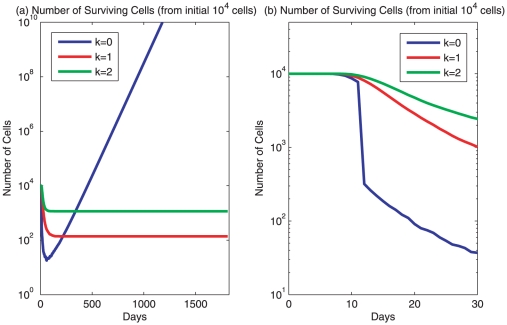
Modeling of cancer cell survival. The metastatic process and the development of clinically detectable metastatic lesions are simulated. The number of cells surviving over the course of a metastatic process is shown. Three masses of 10,000 cells each attempt migration to a distant site, with all cells within a mass possessing either 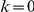, 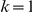, and 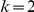, respectively. Every cell has an initial 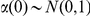 and must pass 5 steps, which are assumed to be negative for any cell to grow. Every step has its specific 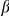 value: dissemination at the primary site 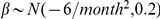 for 3 days, intravasation 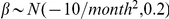 for 2 days, circulation 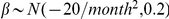 for 1 day, extravasation 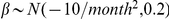 for 2 days, and survival at the ectopic site 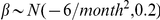 for 3 days. **a.** Cancer cell growth is followed for 5 years to show clinically detectable lesions. **b.** The same cancer cells are followed for only 30 days in order to show the number of surviving cells when they reached distant sites (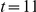 days). The surviving cell number is the summation of all cells whose growth is governed by system 9. No further mutation is considered thus the k value remains constant for each cell and its descendants in the simulations.

**Table 1:** The probability of reduction of the differentiation coefficient to below 0 or 2 by genetic alterations alone, given a frequency of 1 alteration/generation.

Generation	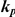	Prob. 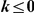	Prob. 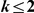
1	1.25	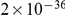	
2	2.09	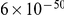	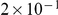
3	2.65	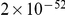	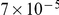
4	3.02	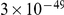	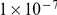
5	3.28	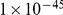	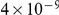
6	3.45	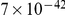	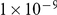
7	3.56	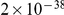	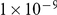
8	3.64	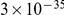	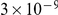
9	3.69	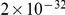	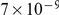
10	3.72	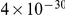	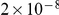
11	3.75	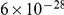	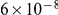
12	3.76	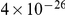	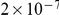
13	3.77	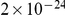	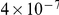
14	3.78	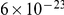	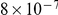
15	3.78	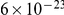	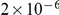

The generation specific 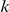 value for a cell is calculated according to the equation 11 and the generation number is derived from equation 5. The accumulated probability for mutations to reduce the generation specific 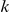 value to 0 or 2 is calculated according to equation 7. Since 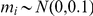 are independent by assumption, the random variable 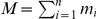 is also normally distributed, with 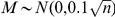. This table shows the probability 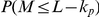, where 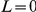 or 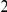.

**Table 2:** Simulation of the effect of mutation rate and environmental factors on the incidence of neoplasm in an individual.

Magnitude	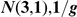	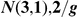	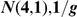	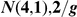
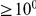	60,000	60,000	60,000	60,000
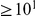	0	0	0.76	87
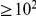	0	0	0	4
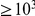	0	0	0	3
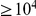	0	0	0	2
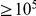	0	0	0	2
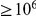	0	0	0	1

As a continuation of Table 1, an individual was assumed to have an incidence of 60,000 cells with 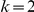 in his life time. These cells were assumed to be in senescent stages (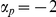) with slight proliferation potential (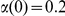). Different mutational rates, 1 (1m/g) or 2 (2m/g) per generation, and varying environmental stimulations, 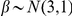 or 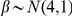 were used to investigate the incidence of escape from senescence and development of different sizes of masses by each of 60,000 cells. Exposure times to chronic stimulation were 6 months every year (Table 2) or all year around (Table 3). Simulations are run for a period of 1 year. Outcome of 60,000 cells with 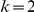 under hormonal exposure for 6 months.

**Table 3:** Simulation of the effect of mutation rate and environmental factors on the incidence of neoplasm in an individual.

Magnitude	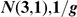	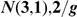	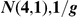	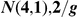
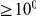	60,000	60,000	60,000	60,000
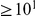	0	0.48	728	3532
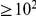	0	0.1	30	869
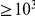	0	0.02	5	424
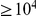	0	0.01	2	286
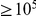	0	0	0.87	215
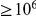	0	0	0.5	164

As a continuation of Table 1, an individual was assumed to have an incidence of 60,000 cells with 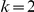 in his life time. These cells were assumed to be in senescent stages (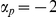) with slight proliferation potential (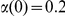). Different mutational rates, 1 (1m/g) or 2 (2m/g) per generation, and varying environmental stimulations, 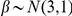 or 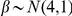 were used to investigate the incidence of escape from senescence and development of different sizes of masses by each of 60,000 cells. Exposure times to chronic stimulation were 6 months every year (Table 2) or all year around (Table 3). Simulations are run for a period of 1 year. Outcome of 60,000 cells with 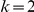 under hormonal exposure for 12 months.

### The clone lifetime of uterine epithelial cells

We first simulated the developmental process of normal epithelial cells in order to understand clone size, the lifespan of every descendant cell, proliferation potential at any moment of development, and more importantly, changes in differentiation coefficient. For the simulations of uterine epithelium proliferation, we set 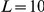, 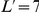, 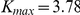, and 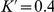 in equations 3 and 4, yielding the following system to describe an individual cell in a clone lifetime: 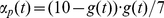
(10)

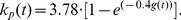
(11)


For the purpose of a more simplified and specific discussion, we will use equations 10 and 11 to substitute for equations 3 and 4 in all of the simulations presented in this paper.

Since endometrial cancer primarily occurs in postmenopausal women, simulations of the normal developmental process in uterine endometrium were conducted either in the absence of estrogenic stimulation or in the presence of low amounts of estrogen exposure. This is represented numerically by either a neutral mean or a small positive mean value for the Gaussian distribution for 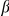, respectively. Numerical simulations, with the specified values for 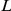, 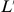, 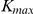, and 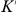, suggest that the possible maximal clone size from a single progenitor cell is about 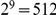 (Fig. 2a). However, when either a neutral or small positive environmental stimulation (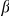) is applied which acts randomly and independently on individual cells every day, cell division in a clone is no longer synchronized due to the differing Î± values for each cell. Peak clone size and time for complete death of a clone vary according to 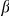 values. In a hypothetical menopausal endometrium without any hormonal effect (zero environment, Fig.2a), a clone will remain at a stable cell number for many years before they enter senescence. With a neutral environment, 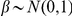 or 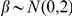, the life cycle of a clone was remarkably shortened although clones took a similar amount of time to reach peak clone size (Fig. 2a). When a positive environment, 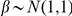 or 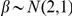, was applied, clone size increased dramatically and the time required to reach the peak clone size and complete senescence were substantially shortened (Fig. 2a).

The values of 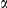 and 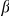 which were set around 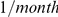 and 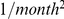, respectively, for a menopausal endometrium may seem too low since many cancer cells double in 1–2 days in culture. However, cancer cells are grown in strong stimulation in vitro with 10–20% fetal bovine serum. Our simulation thus far considers conditions without or with a minimal environmental stimulation. In order to get a sense for what a menstrual cycle will look like in the term of cell proliferation, a strong 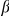, 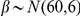 or 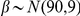 or 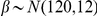, was used to simulate a monophasic oral contraceptive where hormones were given for 21 days (Fig. 2b). The clone size could be 10–1,000 times larger depending upon the hormone levels, consistent with the observation of hyperplasia during proliferative phase of a menstrual cycle. Cell death was sudden when the hormones were withdrawn. However, a clone lifetime was still longer than 28 days since we here did not incorporate any interaction between fast growth of cells and environment, which is simulated in a following section, and the pharmacological effect of sudden hormone withdrawal on blood vessels supporting the endometrial tissues, which is not considered in this manuscript. Once again, the entire endometrial tissue is composed of millions of clones at different stages of their clone lifetime. Every clone starts with zero number and finished with zero number over its entire clone lifetime, with tissue stem cells producing progenitor cells at any moment and resulting in millions of clones at active cycling. The stem cell compartment will not be included in our calculations in this manuscript.

### Malignant transformation during dynamic epithelial cell development

Based on the above description of a clone lifetime, *malignant transformation* (the process of a normal cell becoming a cancer cell), is thus defined on the single cell level as a reduction of a cell's differentiation coefficient 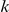 (a process of de-differentiation). This is a definition at the single cell level as opposed to the definition of *tumor* or *cancer* in mass or tissue level. In the traditional definition, a *tumor* is defined as a clinically detected mass, a *cancer cell* is defined morphologically by pathological examination, and *malignancy* as its statistical correlation with patient outcome.

In order to simulate the potential effect of mutations on malignant transformation, one genetic alteration is assumed to occur at every cell division based on data available in the literature [5]. Every alteration occurs randomly and independently, and has an effect on the differentiation coefficient following a Gaussian distribution of 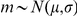. In order to explore the quantitative link between the high incidence of transient and dormant tumors, and the mutation rate and environmental stimulations, we set 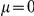 and 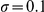, consistent with the hypothesis that mutations could have either a positive or negative effect on the differentiation status, and that most mutations are passenger mutations with little effect.

Accumulation of genetic alterations should be discussed in a setting that a cell has a short lifetime physiologically in an epithelium ranging from days to months but the tissue has billions of cells at any moment and many times more in a human's lifetime. Clearly, a cell accumulates genetic alterations during the developmental process, with a well-differentiated cell (long living with 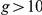) having the most alterations. Using the above values of 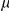 and 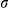, and with the specified values for 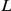, 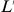, 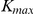 and 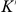, the probability for a single cell to have a 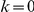 (complete de-differentiation and thus malignant transformation) after 10 generations (11 mutations) is 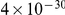 (Table 1). Even assuming 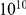 as the total number of cells in an epithelial tissue at any moment, and a one year lifetime of an epithelial clone (a rough estimate for postmenopausal uterine epithelium), a 100 year old woman has statistically no chance to harbor a fully transformed cell through genetic alterations alone. The probability to get a cell with 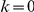 is even smaller in a less-differentiated cell (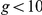) since its accumulated mutational load is small although its 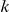 value is low. Although tissue turnover could be as short as several days in some epithelia (e.g., colon crypts), a hundred fold increase in total cell number in an individual's lifetime could not overcome the rare probability of complete malignant transformation by mutation alone. Our model suggests that environmental factors and evolution could play critical roles in the selection and immortalization of partially transformed cells.

### Tumor evolution: growth of a heterogeneous mass consisting of cells with various differentiation coefficients

In order to simulate the evolution of a heterogeneous tumor, we assumed that 4 masses have 1000 cells each at time 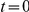. Every cell had an 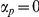 and a starting 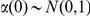. A neutral and slightly varying environmental effect 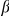 was assumed to follow 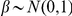. We further assumed the distribution of 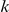 values in a mass followed 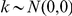, 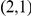, 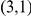 and 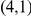, respectively. The median mass with 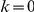 reached 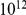 cells (the simulation limit when a simulation was terminated) by 11 months (Fig.3a). The median mass with 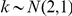 reached the simulation limit by 15 months (Fig.3a). The median size of masses with 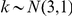 was more than 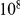 cells by 24 months (Fig.3a). In the contrast, almost all cells in the mass with 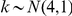 would have a strong resistance potential to maintain an 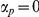 due to high 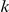 values (a possible least 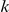 value is about 1 according to the accumulated probability), and a neutral growth stimulation could not produce any significant change of tumor mass (Fig. 3a). While different values for 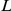, 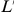, 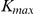, and 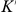 change the times when the simulations reach a simulation limit, the overall dynamics remain the same.

For the mass with the original 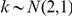, the majority of cells with high 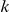 values did not grow much since their resistance potential quickly neutralizes the initial proliferation potentials (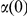) and any growth stimulatory effect from growth signals (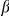) to maintain 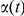 around 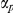 level. It was cells with low 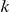 values that lost their capability to maintain 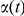 at 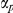 level and multiplied remarkably, contributing increasingly to the population of the mass. The median 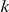 value of the mass declined quickly to reach 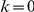 by 6 months (Fig. 3b), indicating that the overwhelming portion of cells were those with 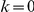. Similarly, the median 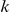 value reached 0.0337 by 21 months for the mass with the original 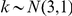, which is approximately the lowest value among the original 1000 cells (Fig. 3b). Comparing to the original mass with 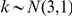, the mass with 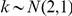 not only had a median 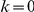 but also the vast majority of cells were 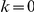 cells by 24 months, forming a dominant subpopulation with growth advantage. The final tumor with a size 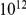 cells appeared to be homogeneous in 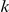 values. These simulations indicate how a cell was selected due to its 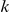 value when a mass grew. The presence of many cells with 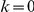 in a mass will provide a potential for unlimited growth, due to the lack of a resistance potential.

This simulation describes one cascade of tumor development and evolution of a dominant subpopulation, where the differentiation coefficient in some cells is already zero (completely transformed). Evolution during the early stage of tumorigenesis is less prominent since the difference of 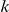 values is small among cells. In this case, environmental factors play an important role in the selection of cells with growth advantage. Therefore, with continuous occurrence of mutations and several cascades of evolution, a substantially transformed cell will emerge and expand as a subpopulation. The entire tumor although still heterogeneous, will become increasingly aggressive. Furthermore, as discussed below, positive and strong stimulations can promote the growth of cells with 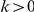, creating a clinically detectable mass consisting of less aggressive cells (with substantially reduced but non-zero 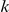 values).

### Calculation of cancer incidences including subclinical (dormant/transient) and clinically detectable tumors

According to the simulation of malignant transformation by genetic alterations in Table 1, the chance of getting a cell with 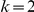 is 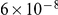, suggesting that 60,000 cells would have a 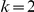 in a human's lifetime with 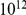 cells. We carried out simulations to test how many cells among those with 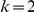 will escape senescence and develop into tumors under certain stimulatory conditions. All cells were already at the senescent stage (defined as 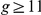), and for further simulations, we assumed an 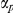 value of -2/month. Hormonal stimulation could be very strong during the menstrual cycle. However, we did not include strong cyclic estrogen but instead included a slightly positive stimulation with 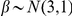 and 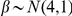 to represent chronic exposure to a weak estrogen in a menopausal woman. Simulations were also performed for various mutational rates. Data shown in Tables 2 and 3 suggest that escape of senescence and development of a mass is positively related to the estrogenic stimulation and mutation rate with the parameter values mentioned earlier. A positive stimulation 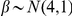 and higher mutation rate (2 mutations per generation) would have 60,000 cells escaping senescence in an individual if the condition lasted for 6 months, including 87 masses with more than 10 cells, 4 masses with more than 100 cells, 3 masses with more than 1,000 cells, 2 masses with more than 10,000 cells, 2 masses with more than 100,000 cells and 1 mass with more than 1,000,000 cells (Table 2). However, if the exposure lasted for one year, the incidence of neoplasm would dramatically increase (Table 3). An individual would have 60,000 cells escaping senescence, 3,523 masses with more than 10 cells, 869 masses with more than 100 cells, 424 masses with 1,000 cells, 286 masses with more than 10,000 cells, 215 masses with more than 100,000 cells and 164 masses with more than 1 million cells. The latter masses would be large enough to be detected in the clinic. When the mutation rate was reduced to one per generation, or stimulation down to 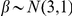 or both, the incidence of tumors with varying sizes was substantially less (Tables 2 and 3). Thus, the incidence of clinical and subclinical forms of neoplasm is affected by mutation rate and exposure to chronic stimulation. If these factors can be quantified, then the incidence of cancer can be predicted for any specific individual. As a general rule, the cancer incidence either subclinical or clinical is positively correlated with an individual's age, hormonal stimulation and mutagenic exposure. This simulation is consistent with reports that subclinical forms of dormant and transient tumors could be commonly present in an individual and the incidence could be much higher if multicentric tumors are included [20]–[23].

### An alternative potential mechanism of the initiation of cancer metastasis: destruction of inter-cellular structure resulting from massive cell death

Since a normal endometrium contains billions of cells, there are multiple clones in different stages of their lifetime providing the tissue a stable total cell number [24]. We hypothesize the existence of many basic tissues. Each of them maintains a stable cell number with a minimal number of developing clones. The maintenance of a stable number of cells in a basic tissue is thus the most fundamental phenomenon of homeostasis at the tissue level [24]. Any sudden increase in cell number (volume) of a tumor will elicit negative reaction from the microenvironment either due to the lack of factors essential for cell survival or due to physical and biochemical disruption of the environment (spatial dependence). Using equations 10 and 11, we arrived at the estimated stable cell number of 18,800 for a basic tissue in the endometrium if a stable cell number was defined as variation of cell number at 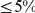 at any time. Obviously, a basic tissue consists of many clones at different stages of their lifetime. To study the interaction between growing cells and their environment, we introduced two concepts: **cellular impact** (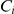) through which a cell exerts its effect on the basic tissue, and **ecological balance** (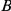) which is the direct reaction from the basic tissue. Ecological balance is a reciprocal action to all cells' impacts (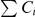) on a basic tissue.

For any individual cell, the instantaneous impact of the cell on the basic tissue at time 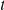 is proportional to its rate of growth, 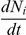 (equation 2), so
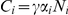
for some proportionality constant 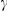. Summing over all 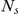 cells, 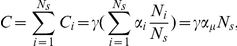
where 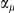 is the expected value of the 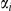
's. In a basic tissue, there is a stable cell number, so it should hold that 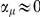. It follows that 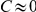 in a basic tissue.

Ecological balance will not act on a specific cell as a direct reaction to its 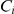 value, but will instead affect all cells in the basic tissue in a stochastic fashion following a Gaussian distribution using 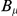 and 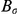 values. Thus, 
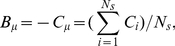
(12)

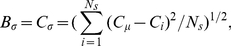
(13)


In order to simulate a potentially negative effect from the microenvironment on a fast growing tumor and investigate the effect of differentiation coefficients on the growth, we conducted simulations of four neoplastic cells with 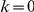, 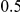, 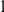 and 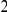 arising in four separate basic tissues. They grew under identical conditions with 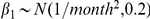 and an initial 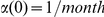. The environmental reactions to tumor growth, 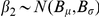 were calculated according to Equations 12 and 13, and assigned to every surviving cell every day randomly and independently. Our results showed that for the first 12 months, the cell with 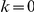 proliferated very fast and reached the median mass size of approximately 170,000 cells by 5 months (Fig. 4a). A cell death of more than 6000 cells occurred by 5 months (Fig.4b), which was followed by a slow increase in mass size but accompanied by increasing cell death. More than 7 million cells died while the mass size reached only 680,000 cells by 12 months (Fig.4a and 4b), suggesting that the number of cell deaths is more than 10 times the mass size. The cell with 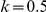 proliferated and reached the similar mass size by 12 months (Fig.4a). However, significant cell death did not occur until the 9th month (Fig. 4b). The two cells with 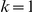 and 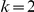 grew very slowly (Fig.4a) and did not have any substantial cell death by 12 months (Fig. 4b).

The above simulations suggest an interesting possibility. Massive cell death causes destruction in the tissue, resulting in widespread cell relocation and rupture of blood vessels. Thus, we speculated that massive cell death could lead to cell motility and tumor expansion. Simulations conducted by Enderling et al suggests a similar role of cell death for tumor expansion and invasion [19]. The simulations here showed that the faster proliferation of a more aggressive tumor with lower k cells did not necessarily lead to an overtly larger tumor but instead might result in earlier tissue invasion and metastasis. This is consistent with clinical observations that an aggressive cancer does not often present as a solitary large primary mass but a benign tumor does. As indicated by the simulations, tissue invasion and metastasis might occur in the fifth month when the mass size of k = 0 tumor was approximately 170,000 cells.

However, the simulations above did not consider the positive interactions between cancer cells and microenvironment. Cancer cells may release some factors to enhance angiogenesis, and the surrounding tissues may adjust to accommodate the growing mass. These positive interactions may catch up to the increase in tumor size and reach a new balance for further tumor growth. In addition, a slowly proliferating tumor may have a good chance to reach a new balance and achieve a larger tumor size without metastasis.

### Survival of metastatic cells in the circulation and development of clinically detectable metastatic lesions

We now investigate the likelihood of cancer cell survival during the metastatic process and the role of the differentiation coefficient in the establishment of metastatic lesions. We assume that a cancer cell takes 5 steps to reach an ectopic (distant) site and attain proliferation for the establishment of a metastatic lesion according to the main steps in the formation of a metastasis [25]. Each step is rate-limiting and modeled as negative growth stimulation. Three masses each had 10,000 cells migrating to a distant site and their 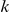 values for every one of 10,000 cells were 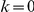, 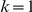, and 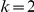, respectively (Fig. 5). The implemented 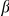 values for each rate-limiting step are: 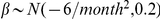 representing dissemination at the primary site for 3 days, 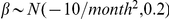 representing intravasation for 2 days, 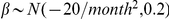 representing circulation for 1 day, 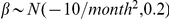 representing extravasation for 2 days, and survival at the ectopic site with 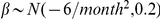 for 3 days, following a similar schedule in experiments [26]. Metastasis is assumed to be followed by a 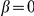 for 5 years without distinguishing favorable and unfavorable sites. Cells from the three masses have shown a striking difference in their survival and establishment of metastatic lesions. The survival rate at the end of the metastasic cascade (after 11 days) is similar among the three masses with more than 90% survival (Fig. 5a and 5b), consistent with experimental data showing that majority cells survive the metastatic process [26], [27]. However, the mechanism for metastatic inefficiency is different among cells with different levels of malignancy. A more malignant cell (with 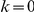) was more vulnerable to the harsh conditions during metastasis and had the lower survival rate (Fig. 5b). Only 19 (0.19%) out of 10,000 cells with 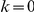 survived, consistent with *metastatic inefficiency* primarily due to subsequent cell death resulting from apoptosis [28], [29]. The surviving rates for cells with 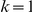 and 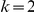 were 1.3% (132 out of 10,000) and 11.8% (1179 out of 10,000), respectively (Fig. 5b). But none of them could retain sufficient growth to develop into a clinically detectable metastatic lesion over 5 years (Fig.5a), which is consistent with the mechanism of metastatic inefficiency due to failure of subsequent growth [26]. Only those cancer cells with 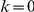 could grow into a metastatic lesion after a successful metastasis (Fig. 5a). Cells with 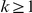 remained dormant without further mutations and environmental stimulations. Thus, establishment of clinically significant ectopic lesions correlates positively with the number of cells with extremely low 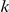 value in a primary tumor and the fast growth of the primary tumor (Fig. 4), but negatively with the extent of harsh conditions in the metastatic process (Fig.5). This result is consistent with the well-established role of metastatic lesions to predict patient outcome. But our model provides an approach to quantitatively and prospectively predict the chance of a metastatic lesion through analysis of the primary tumor, and may be particularly useful to predict the existence of micrometastasis. For cells with 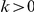 to grow in ectopic sites, a positive 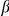 is required. The value of 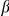 determines whether an ectopic site is favorable for the development of metastatic lesions. Thus, the long-held hypothesis of *seed and soil* for pathogenesis of metastasis [25], [29] is quantitatively modeled here.

These results are also consistent with clinical observations that metastatic endometrial cells are not cancer cells in endometriosis. Endometrial cells undergo a fast proliferation during the early menstrual cycle due to estrogenic stimulation and massive cell death occurs late in the cycle due to withdrawal of hormones. Some endometrial cells are transported through fallopian tubes and implanted on the surface of the pelvic cavity. These endometrial cells survive the metastatic process and the condition in ectopic sites due to their high differentiation coefficient. As with cells in the primary site, ectopic endometrial cells only proliferate in response to high estrogen. Large 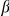 is derived from high estrogen at the proliferative phase of a menstrual cycle, similar to those modeled at Fig. 2b. The ectopic endometrial tissues become atrophic with prolonged progestin treatment, pregnancy and at menopause. Thus, the occurrence of endometriosis is due to 1) successful metastasis due to mechanical transportation of endometrial tissues into ectopic locations; 2) high survivability of cells due to their high differentiation coefficient; and 3) periodic proliferation stimulated by strong and cyclic hormones. Most important of all, these ectopic endometrial cells have high k values and are not cancer cells.

## Discussion

It has been the hope of every cancer researcher that complex cancer biology can be rationalized based on a small number of underlying principles which govern the myriad cancer genotypes and phenotypes [3], [30]. The exceptional elucidation of the general principles of cancer formulated by Hanahan and Weinberg, here referred to as the W theory [6], coupled with efforts to elucidate the mechanisms leading to carcinogenesis exemplified by the seminal work of Vogelstein and colleagues, here referred to as the V theory [4], [5], combine to provide a solid heuristic foundation, referred to here as V/W. Our model, using simple mathematical equations featuring a small number of variables, is an attempt to quantitatively and prospectively interpret V/W. It is also an attempt to develop a classical *insight and theory* approach wherein principles and equations are derived from fundamental concepts and deductions as opposed to a direct a priori analysis of data followed by an a posteriori explanatory model.

Assuming in our model that cells targeted for malignant transformation include well-differentiated cells, malignant transformation can be considered to be a process of de-differentiation [17], which can be quantitatively described by a differentiation coefficient. As illustrated at Fig. 1, numerous mutations occur in a tissue with billions of cells constantly turning-over. Most non-stem uterine epithelial cells have a lifespan limited to days or months. The cumulative effect of mutations is neutral and not transformative in vast majority of normal cells since the mutational effect follows a Gaussian distribution as proposed by our model. One out of billions of cells acquires a mutation, or several mutations, which result in de-differentiation, which is captured in a differentiation coefficient, 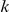 in our model (equation 7). A reduced 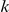 value results in a lower resistance potential (equation 6), which will allow the cell to multiply in a subnormal level of hormonal (or other) stimulation (system 9) and create a population of cells well beyond homeostasis that might initially be hyperplastic or benign. Additional mutations can further reduce cells' 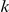 values yielding selective fitness and a dominant sub-population due to their lower 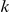 values (Fig.3). A lower 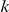 value corresponds to further de-differentiation thus further malignant transformation. Again, some cells in this population will emerge with even lower 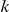 values and continue the cascade of malignant transformation and tumor evolution. This mathematical expression of multistage oncogenesis is strikingly complementary to the V theory [4], [5]. Additionally, as simulated in the Fig. 4, rapid growth of an increasingly malignant cell population will be accompanied by massive cell death thus creating a potential mechanism of metastasis that does not require but facilitate further mutations. This result provides a quantitative interpretation of Jones' genomic analysis [5] opposing the long held paradigm that the appearance of metastatic cell populations is driven by additional mutations [4], [5].

The V theory of tumorigenesis posits the fundamental role of genetic mutations in malignant transformation as the pathway for tumor progression. While it is likely that only a small number of mutations are driver mutations and the rest are passengers, our model accounts for the contribution of every potential mutation in a normal distribution and, from this, the collective roles played by all mutations can be quantified. The precise contribution of a specific mutation has to be analyzed in patient samples [12]. Sequencing of many tumor genomes is increasingly being realized with the initiation of Cancer Genome Altas and Cancer Genome Anatomy project.

Tumor evolution is described by many investigators analogously to Darwinism with tumors being a clonally-derived cell populations and progression the result of some cells acquiring advantages through mutations [31], [32]. The descriptive theories are complemented by our quantitative analysis that has a potential to interpret a wide range of different cancer phenotypes. Use of our model either in population level or single cell level may prove useful in the reconciliation of some seemingly contradicting observations. Advancing age is the most potent of all carcinogens [33]. On the other hand, cellular senescence (ageing) is as potent as apoptosis in suppressing spontaneous tumorigenesis [34]. In our model the probability of malignant transformation increases significantly as generations progress at the single cell level. However, senescence significantly increases when generation progresses (equation 3). These factors play against each other and the balance will be shifted to favor transformation if environmental stimulation increases to escape senescence by providing cell proliferation beyond intrinsic potential. At the tissue level, the probability of emergence of a malignant cell will increase with age since an older individual will have more cell cycles in their lifetime and, consequently, increased cancer incidence.

Our model uses cellular generations as a biologic clock which is a fundamental variable in determining cellular proliferation and differentiation. While cell division is intuitively simple and clear from a morphological perspective, a mathematical expression is necessary to provide a usable description of propagation of an individual cell during a clone lifetime. Cell divisions are determined by proliferation potential that depend on proliferation history and specific factors, such as mutations and environmental cues. This approach allows us to follow the time course of a cell during a clone lifetime without a need for counting cell divisions experimentally, therefore measuring intra-tumor heterogeneity at any time quantitatively. A proposed generation limitation is a quantitative interpretation of replicative senescence.

Our model simultaneously describes individual cells as well as cell populations (or tumors), thus preserving population heterogeneity. This approach captures qualitative change in individual cells as the critical event for tumor progression (cascade of tumor evolution). This additive approach to summarizing individual cells may allow us to prospectively describe incidences of dormant/transient cancers (those cancers either not easily diagnosed clinically or not diagnosed at all [20], [23]). In the future, detailed comparison between lethal cancer and non-disease cancer will be a much more informative way in which to identify truly causal genetic alterations. In a normal physiologic scenario, growth of a clone or an individual cell of the clone will have minimal effect on the environment. However, growth of a malignant mass is fast and can be extensive, exceeding normal physiological limits. Thus, the impact of the mass on its surroundings will be significant and, conversely, the reaction of the surrounding environment will also be dramatic (equation 12). This will cause massive cell death (Fig. 4), here identified as a potential mechanism of the initiation of metastasis.

Our model is best described as a classic theoretical model of *insight and theory* approach and the coherence of the model is fully embedded in the mathematical equations. It is a novel approach and still at an early stage of development. Numerous tissue- and cell- specific variables have to be quantitatively determined before such a model can be experimentally applied to a wide range of cancer phenomena. Modeling of environment effects in this model also will require modification over time. Fortunately, there are many excellent models regarding the role of environment factors in oncogenesis and tumor progression [35]–[39]. Our hope is that we may be able to integrate our model with those existing models. Such a theoretical approach should be complimentary to empirical and bioinformatic approaches to cancer research in a way that data are used iteratively to test and update equations and laws.

Taken together, our modeling of malignant transformation and tumor progression, based on a few assumptions and a few mathematical equations, is capable of comprehensively describing major cancer phenotypes and has suggested some interesting features for neoplasm: 1) incidence of neoplasm is much higher than what is diagnosed in clinics due to the frequent existence of dormant/transient neoplasm; 2) neoplastic cells are heterogeneous in a tumor and the aggressive cells (with low differentiation coefficients) have a growth advantage that allows them to dominate the tumor during its evolution; 3) rapid proliferation can result in cell death and lead to cell dislocation and metastasis; 4) more aggressive cancer cells have less chance to survive metastasis but are more capable of growing into a clinically significant lesion. Therefore, an aggressive tumor, defined by the presence of cancer cells with low differentiation coefficients, will have a poor patient outcome since the significant number of 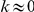 cells will ensure fast growth in the primary and ectopic sites in most environmental conditions.

## Methods

### The Model

In this paper, we discuss a fundamental model for cancer that has shown the capability to systematically capture many varied cancer phenotypes. The quantitative interpretation of oncogenesis is preceded by a comprehensive description of normal tissue regeneration in uterine epithelium, based on the assumption that every one of billions of normal cells follows an inherent program for proliferation and differentiation through a cellular interpretation of chronological time. The landscape of physiological tissue regeneration provides a picture of normal cell heterogeneity and dynamics, and serves as the reference for tumor development resulting from genetic insults and environmental stimulations. It is a single-cell based model, where the evolution of each tumor is described in terms of the evolutionary dynamics of its constituent cells. The quantitative basis of the model is a quantification of a cell's differentiation status, a measurement of how “normal” a cell is, and how this status varies due to different mutations. The cellular dynamics are defined on a per cell basis by the system of equations: 
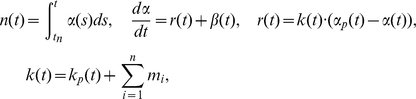
where 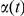 is a cell's proliferation potential at time 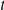 and 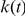 is its differentiation coefficient. The parameters 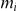 are the mutational effects on the differentiation coefficient, and are distributed according to a Gaussian distribution with mean 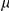 and standard deviation 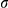, 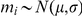. The parameter 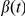 is the environmental effect and is considered as an overall effect over the course of one day, and is also chosen from a Gaussian distribution. Each cell has associated with it certain programmed levels of proliferation potentials, 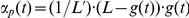, and differentiation coefficients, 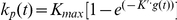, which are dependent on the maturity of the cell, measured by its generation number, 
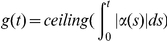
. The resistance potential (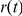) is a restorative force, acting to restore 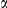 back to its programmed level. A measure of the cell's status toward either dividing or dying is provided by 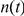, where the cell was born at time 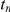. This system of equations is valid over the lifetime of the cell, that is, from the time it is born (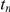) until it either dies (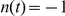) or divides (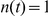). In the case that the cell divides, the two daughter cells inherit 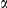, 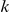, and 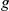 from the parental cell. Each cell then proliferates independently of the other according to this system of equations. The developmental process continues with cell-specific 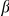
's, and the mutations accumulated by each daughter cell are independent of those accumulated by the other daughter cell.

In all of the simulations presented in this manuscript, we consider the model case where 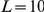, 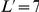, 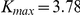, and 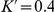, so that the programmed proliferation potentials and differentiation coefficients are given by 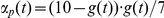 and 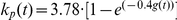 respectively. In all cases considered in this manuscript, we arbitrarily take 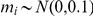. However, the frequency of these mutations and the distributions for the environmental effects vary among the experiments considered.

Each simulation is performed using the computer programming language Fortran 90, with the resulting graphics generated with Matlab. The time-step for each simulation is 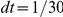. Over the course of one time-step, the system can be solved explicitly, with an algorithm outlining this process provided as Supporting Information S1. At time 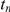, a 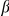 is chosen for each cell, and 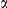, 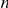, and 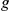 are advanced over 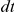. If the cell advances in generation number (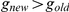), the cell gains a number of mutations, dependent on the experiment. If 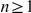, the cell has proliferated, with the (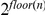) daughter cells inheriting the parent's 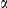 and 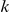. If 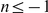, then the cell is considered dead and is removed from the simulation.

### The clone lifetime of uterine epithelial cells

In this section, we consider two experiments. The first experiment, illustrated by Fig. 2a, is an analysis of the clone size in uterine epithelium when there is little or no estrogen exposure. A 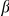 value is assigned to an existing cell every day, randomly and independently. Four different trials are considered: 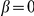, 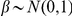, 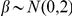, and 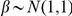. 101 simulations were performed for each trial, with Fig. 2a illustrating the median curves for each trial, and the maximal, 75th percentile, the median, 25th percentile, and the minimal sizes of clones are presented in Fig. S1.

The second experiment, illustrated by Fig. 2b, is an analysis of the clone size in uterine epithelium with the presence of monophasic contraceptives. The exposures to large concentrations of estrogen are represented here by strong and positive 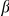
's. Three trials are considered: 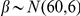, 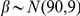, and 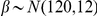. These environmental effects are applied for 21 days before being removed. As in the previous figures, 101 simulations were performed for each trial and the median clone sizes are presented. All experiments are performed in the absence of mutations. The maximal, 75th percentile, the median, 25th percentile, and the minimal sizes of clones are presented in Fig. S2.

### Malignant transformation during dynamic epithelial cell development

According to this model, the differentiation coefficients are altered solely due to mutations. In this section, we consider the accumulated probabilities of a cell to attain a differentiation coefficient of 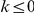 or 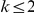 after 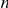 generations. We assume that one mutation occurs per generation, with each mutation being random and independent, with 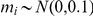. Since the mutations are independent by assumption, the random variable 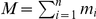 is also normally distributed, with 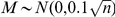. Table 1 shows the probability 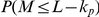, where 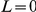 or 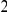.

### Tumor evolution: growth of a heterogeneous mass consisting of cells with various differentiation coefficients

In this section, the evolution of a mass comprising cells with various differentiation coefficients is explored. Four separate trials are considered. Each trial begins with 1000 cells that have reached maturity, that is, 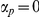. Each cell is assigned a proliferation potential with 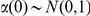. Every cell was assigned a 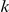-value from either 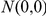, 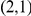, 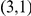 and 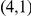, depending on the trial. Only nonnegative 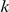 values are considered in this paper, so if 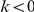, we set 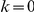. No mutations were considered, and therefore each cell and its descendants retained their initial 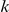-value. For each cell, the environmental effects were chosen with a neutral mean, 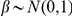. The simulations were performed until either a simulation period of 2 years had passed or the total cell number had reached 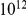. Fig. 3a represents the median mass sizes of 101 simulations among each of the four trials, while Fig. 3b illustrates the evolution of the median 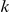-values of the cells within the median masses of each trial. The maximal, 75th percentile, the median, 25th percentile, and the minimal values of mass sizes and 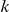 values are presented in Fig S3 and S4, respectively.

### Calculation of cancer incidences including subclinical (transient and dormant) and clinically detectable tumors

In Table 1 it is shown that, for the test case, the probability of a specific cell reaching 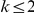 due solely to mutations by the time it reaches programmed senescence, that is 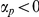 after 11 generations, is 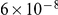. Assuming that there are roughly 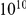 epithelial cells at any time, each with a lifespan of 1 year, a human with a lifetime of 100 years would have 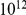 epithelial cells throughout their lifetime, with roughly 60,000 cells having 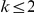 due solely to mutations. In this section, we analyze the evolution of these 60,000 cells to calculate empirical probabilities of having masses of various orders of magnitude during a lifetime. Two separate experiments are considered, each with four separate trials. In both experiments, we begin with 60,000 cells, each with 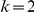. The cells begin at the senescent stage, with 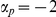. However, their actual proliferative potential is 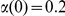. In the first experiment, there is an environmental effect present for six months every year, while in the second experiment, the environmental effect is present year-round. The four trials for each experiment have either one or two mutations per generation, and an environmental effect following 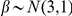 or 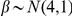. For each trial, 101 simulations are performed, and empirical probabilities are calculated from the data generated. The mean number of tumors of various sizes that can be expected among the 60,000 cells are illustrated when the probability of having at least one tumor of each magnitude was one. In the case that a tumor cannot be probabilistically guaranteed, the probability of having at least one tumor of each magnitude is illustrated.

### An alternative potential mechanism of the initiation of cancer metastasis: destruction of intercellular structure resulting from massive cell death

In this section, the reaction of the microenvironment surrounding the tumor is included into the model as an additional environmental effect. The impact of a cell on its environment (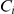) is proportional to its rate of growth 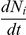, so 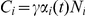. The total environmental impact of a tumor is then 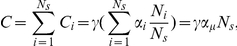
where 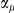 is the expected value of the 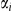
's. The environmental response to this cumulative force acts on each cell in a stochastic fashion, with mean 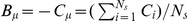 and standard deviation 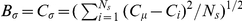. Fig. 4 illustrates the outcomes of four trials. Each trial begins with one cell. A weak growth stimulation following 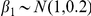 is chosen, with this value being the initial environmental effect for all four trials. Their initial proliferation potentials are all identically 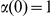. However, their initial differentiation coefficients differ, with either 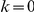, 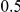, 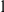, or 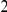. No mutations are considered, so these 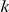-values remain constant among all descendant cells. At each time step, a new growth stimulation 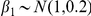 and environmental reaction 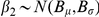 are calculated. One hundred and one simulations were performed for each trial, and Fig. 4 illustrates the median curves for the mass sizes and the accumulated number of cell deaths occurring for each trial.

### Survival of metastatic cells in the circulation and development of clinically detectable metastatic lesions

In this section, the likelihood of cancer cell survival during the metastatic process is explored, along with the role of the differentiation coefficient in the establishment of a metastatic lesion. The cancer cells are assumed to take five steps to reach an ectopic site and establish a metastatic lesion. The five steps are represented by negative growth stimulations. Three trials are considered. Each trial begins with 10,000 cells. The 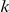-values of all 10,000 cells are either 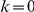, 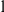, or 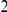, depending on the trial. No mutations were included, so all descendants retained the same 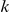-value. Their initial proliferation potentials are chosen from 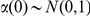. Each cell underwent the following environmental effects over the course of the five steps: 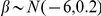 for 3 days representing dissemination at the primary site, 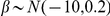 for 2 days representing intravasation, 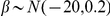 for 1 day representing circulation, 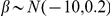 for 2 days representing extravasation, and 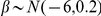 for 3 days representing survival at the ectopic site. This was followed by an environmental effect of 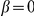 for 5 years. 101 simulations were performed for each, with the median curves for the number of surviving cells illustrated in Fig. 5. The maximal, 75th percentile, the median, 25th percentile, and the minimal values of mass sizes are presented in Fig S5.

## Supporting Information

Figure S1
**A clone lifetime of uterine epithelial cells during menopause, illustrated by change of cell number of the clone over time.** 101 simulations are performed for all cases, with the following trajectories presented here: minimum, 25th percentile, median, 75th percentile, and maximum. (a) Results for 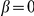. (b) Results for 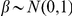. (c) Results for 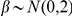. (d) Results for 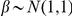. (e) Results for 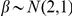.(EPS)

Figure S2
**A clone lifetime of uterine epithelial cells with oral contraceptives, illustrated by change of cell number of the clone over time.** 101 simulations are performed for all cases, with the following trajectories presented here: minimum, 25th percentile, median, 75th percentile, and maximum. (a) Results for 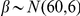. (b) Results for 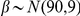. (c) Results for 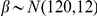.(EPS)

Figure S3
**Simulations of tumor evolution: size change of intra-tumor subpopulations of tumors with heterogeneous cells.** The growth curves of four masses with 1000 heterogeneous cells each, specified by different differentiation coefficients to indicate the loss of differentiation. 101 simulations are performed for all cases, with the following trajectories presented here: minimum, 25th percentile, median, 75th percentile, and maximum. (a) Results for 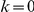. (b) Results for 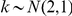. (c) Results for 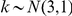. (d) Results for 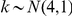.(EPS)

Figure S4
**Simulations of tumor evolution: redistribution of k-values among intra-tumor subpopulations of tumors with heterogeneous cells.** Change of the median value of differentiation coefficients with respect to time for the masses presented in Figure 3. (a) Results for 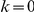. (b) Results for 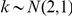. (c) Results for 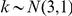. (d) Results for 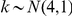.(EPS)

Figure S5
**Modeling of cancer cell survival during metastasis and the establishment of metastatic lesions.** 101 simulations are performed for all cases, with the following trajectories presented here: minimum, 25th percentile, median, 75th percentile, and maximum. (a) Results for 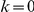. (b) Results for 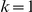. (c) Results for 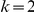.(EPS)

Supporting Information S1 **Computational Algorithm and Additional Figures for “Quantitative Interpretation of a Genetic Model of Carcinogenesis Using Computer Simulations”.**
(PDF)
