# Supplementary material for: Quantitative Interpretation of a Genetic Model of Carcinogenesis Using Computer Simulations
Source: PLoS One. 2011 Mar 8;6(3):e16859. doi: 10.1371/journal.pone.0016859 (PMC3050823; doi:10.1371/journal.pone.0016859)
Supplement: Supporting Information S1 — Computational Algorithm and Additional Figures for “Quantitative Interpretation of a Genetic Model of Carcinogenesis Using Computer Simulations”. (PDF) [file pone.0016859.s006.pdf]

Supporting Information - Computational Algorithm and Additional Figures for "Quantitative Interpretation of a Genetic Model of Carcinogenesis Using Computer Simulations"

Donghai Dai[1*], Brandon Beck[1], Xiaofang Wang[1], Cory Howk[1], Yi Li[2]
1 Dept. of Obstetrics & Gynecology, University of Iowa, Iowa City, Iowa, USA
2 Dept. of Mathematics, University of Iowa, Iowa City, Iowa, USA
∗ **E-mail: donghai-dai@uiowa.edu**

Introduction

In the following figures we present several trajectories for simulations of the stochastic model. For each experiment, 101 simulations were performed. The trajectories were ranked by cell number within the mass at the time the simulations were terminated, with two exceptions. Some of the masses disappeared (cell number = 0) before termination. These masses are ranked by the order in which they disappeared. Some of the mases grew above a preset limit before the time interval of interest elapsed. These masses are ranked by the order in which they surpassed the preset limit. The trajectories presented in this supplemental correspond to those at rank 1, 25, 51, 76, and 101. That is, they represent the minimum terminal result, maximum terminal result, the median, and the results at the 25th and 75th percentiles.

Results

Flowchart for the evolution of a cell by the stochastic model.

The following step-by-step algorithm can be used to advance the cell number over time. This is the algorithm for the advancement of one cell by one time step Δt. This would have to be repeated for each cell in the system. Over the course of one time step (Δt), the system is not stochastic, and therefore the differential equation for $\alpha(t)$ can be solved explicitly. We can then derive expressions for the advancement of $N^{(exp)}(t) = \int_{t_0}^{t} \alpha(s)ds$ where t_0 is the time of birth of the cell, $N(t) = 2^{N^{(exp)}(t)}$, $I(t) = \int_0^t |\alpha(t)|dt$, and $g(t) = ceiling\Big(I(t)\Big)$. Let p denote the number of mutations gained per generation.

1. $t_{new} = t_{old} + \Delta t$
2. Assign β.
3. Evaluate cellular status:

- If $k \neq 0$:
  a) Define $C = \alpha_{old} - (\alpha_p + \frac{\beta}{k})$.
  b) $\alpha_{new} = (\alpha_p + \frac{\beta}{k}) + Ce^{-k\Delta t}$
  c) $N^{(exp)}_{new} = N^{(exp)}_{old} + (\alpha_p + \frac{\beta}{k})\Delta t + \frac{C}{k}(1 - e^{-k\Delta t})$
  d) Is $0 < \frac{-1}{C}(\alpha_p + \frac{\beta}{k}) < \infty$ and $\widehat{\Delta t} = \frac{-1}{k} \ln\left(\frac{-1}{C}(\alpha_p + \frac{\beta}{k})\right) \in (0, \Delta t)$?

  – If 'yes', then

$$I_{new} = I_{old} + |(\alpha_p + \frac{\beta}{k})\widehat{\Delta t} + \frac{C}{k}(1 - e^{-k\widehat{\Delta t}})| + |(\alpha_p + \frac{\beta}{k})(\Delta t - \widehat{\Delta t}) + \frac{C}{k}(e^{-k\widehat{\Delta t}} - e^{-k\Delta t})|.$$

  – If 'no', then $I_{new} = I_{old} + |(\alpha_p + \frac{\beta}{k})\Delta t + \frac{C}{k}(1 - e^{-k\Delta t})|$.

- If $k = 0$:
  a) $\alpha_{new} = \alpha_{old} + \beta\Delta t$
  b) $N_{new}^{(exp)} = N_{old}^{(exp)} + \alpha_{old}\Delta t + \frac{\beta}{2}(\Delta t)^2$
  c) Is $\beta \neq 0$ and $\widehat{\Delta t} = \frac{\alpha_{old}}{\beta} \in (0, \Delta t)$?

  – If 'yes', then

$$I_{new} = I_{old} + |\alpha_{old}\widehat{\Delta t} + \frac{\beta}{2}(\widehat{\Delta t})^2| + |\alpha_{old}(\Delta t - \widehat{\Delta t}) + \frac{\beta}{2}\Big((\Delta t)^2 - (\widehat{\Delta t})^2\Big)|.$$

  – If 'no', then $I_{new} = I_{old} + |\alpha_{old}\Delta t + \frac{\beta}{2}(\Delta t)^2|$.

- $g_{new} = ceiling(I_{new})$

- $N_{new} = 2^{N_{new}^{(exp)}}$

4. Is $g_{new} > g_{old}$?

- If 'yes', then update α_p and k_p. If $N_{new}^{(exp)} < 1$, then also update k by $k_{new} = k_{old} + \sum_{i=1}^{p\cdot(g_{new}-g_{old})} m_i$, $m_i \sim N(0, 0.1)$. If $N_{new}^{(exp)} \geq 1$, then wait to update k with mutations until step #9.

5. Is $N_{new}^{(exp)} < -1$?

- If 'yes', the cell dies.
- If 'no', continue.

6. Is $N_{new}^{(exp)} \geq 1$?

- If 'yes', then continue to #7.
- If 'no', then return to #1.

7. The cell has proliferated into $2^{floor(N_{new}^{(exp)})}$ daughter cells. Replicate $2^{floor(N_{new}^{(exp)})} - 1$ new cells.
8. Assign each daughter cell α, g, I, k, α_p, and k_p from the parent cell.
9. For each cell:

- If $g_{new} > g_{old}$, then for each of the $2^{floor(N_{new}^{(exp)})}$ daughter cells, individually update k for each cell with mutations by $k_{new} = k_{old} + \sum_{i=1}^{p\cdot(g_{new}-g_{old})} m_i, \quad m_i \sim N(0, 0.1)$.

- $N_{new}^{(exp)} = N_{new}^{(exp)} - floor(N_{new}^{(exp)})$.

10. Return to #1 for each daughter cell, and run the algorithm for each separately.

Note, however, that as we proceed through the algorithm in practice, we do not need to explicitly keep track of the values of $N(t)$, $g(t)$, $\alpha_p(t)$, or $k_p(t)$.

The clone lifetime of uterine epithelial cells during menopause.

In this experiment, we examine the effect of microenvironmental stimulation on normal clonal growth. We begin with one cell, with initial conditions

$$g(0) = 0, \qquad \alpha_p(0) = 0, \qquad k_p(0) = 0, \qquad \alpha(0) = 1.$$

Experiments are performed with sustained $\beta = 0$, $\beta \sim N(0,1)$, $\beta \sim N(0,2)$, $\beta \sim N(1,1)$, and $\beta \sim N(2,1)$ in the absence of mutations. Results are shown in Figure S1. There does not appear to be much deviation from the median behavior. However, it is clear that β can have the effect of increasing mass size, but shortening its lifetime.

The clone lifetime of uterine epithelial cells in the presence of oral contraceptives.

In this experiment, we examine the effect of hormonal stimulation on normal clonal growth, a situation mimicking that occuring via oral contraceptives. We begin with one cell, with initial conditions

$$g(t) = 0, \qquad \alpha_p(0) = 0, \qquad k_p(0) = 0, \qquad \alpha(0) = 1.$$

Experiments are performed with $\beta \sim N(60,6)$, $\beta \sim N(90,9)$, and $\beta \sim N(120,12)$ applied for 21 days, followed by sustained $\beta = 0$. All experiments are performed in the absence of mutations. Results are shown in Figure S2. There again does not appear to be much deviation from the median behavior. However, β can have a massive effect on overall mass size.

Tumor evolution: growth of a heterogeneous mass consisting of cells with various differentiation coefficients.

In this experiment, we examine the evolution of a heterogeneous mass composed of mature cells. Clone size is illustrated in Figure S3. The median k-values of these masses are shown in Figure S4, illustrating the evolutionary advantage of cells with lower k-values. We start with a mass of 1000 cells, with initial conditions

$$\alpha_p(0) = 0, \qquad \alpha(0) \sim N(0,1).$$

Experiments are performed with $k = 0$, $k \sim N(2,1)$, $k \sim N(3,1)$, and $k \sim N(4,1)$. All cells are exposed daily to $\beta \sim N(0,1)$. If $k < 0$ for some cell, we reassign it $k = 0$. Experiments are performed in the absence of mutations and without advancing generations ($g(t)$). We can see from Figure S3 that there is much deviation from the median result, either in the cell number after two years, or the rapidity with which the masses approach the simulation limit of 10^{12} cells. For mass sizes above 10^7, we approximate the size by setting $\beta = 0$ and analyzing the resultant system of differential equations for clone growth. We can see from Figure S4 that cells with lower k-values have an evolutionary advantage ($\frac{d\alpha}{dt} = -k\alpha + \beta$) and are selected for as the mass progresses. Note that the masses that reached the simulation limit size of 10^{12} cells had median $k = 0$.

Survival of metastatic cells in the circulation and establishment of metastatic lesions.

In this experiment, we examine cells undergoing the process of metastasis. We represent the general steps of the dislocation process with microenvironmental effects (β) representative of the relative difficulty of

each step. We start with 10,000 cells, with initial conditions

$$\alpha_p(0) = 0, \qquad \alpha(0) \sim N(0,1).$$

Experiments are performed with $k = 0$, $k = 1$, and $k = 2$. Cells are exposed to

$\beta \sim N(-6, 0.2)$ for 3 days representing dissemination at the primary site,
$\beta \sim N(-10, 0.2)$ for 2 days representing intravasation,
$\beta \sim N(-20, 0.2)$ for 1 days representing circulation,
$\beta \sim N(-10, 0.2)$ for 2 days representing extravasation,
$\beta \sim N(-6, 0.2)$ for 3 days representing survival at the primary site.

This is followed by $\beta = 0$ for 1800 days. Experiments are performed in the absence of mutations and without advancing generations ($g(t)$). Results are shown in Figure S5. We can see from the figure that there does not seem to be much deviation from the median behavior. However, we can see that, although higher k-values permit cells to survive the metastasis to the ectopic location, it is the cells with lower k-values that thrive in the new environment, producing masses.

Discussion

One observation from these simulations is that, although the environmental stimulation β can have a large effect on mass size, it is a consistent effect. That is, similar cells have similar outcomes; there is not much variation from median behavior. However, as we can see from Figure S3, variations in k can lead to wildly different behaviors among similar cells in similar environments. This dynamic between the effects of variations in β and k in this mathematical model may lead to many interesting results. Therefore, one point of future research will be to provide a global sensitivity analysis of the mathematical model along with biological interpretations of the results.
